# Contribution of pharmacist intervention to postoperative nausea and vomiting prophylaxis in routine multidisciplinary practice: a retrospective observational study

**DOI:** 10.1186/s40780-026-00590-2

**Published:** 2026-06-01

**Authors:** Kotaro Sato, Shinya Suzuki, Takuma Koinuma, Akinori Omata, Emiko Iguchi

**Affiliations:** 1 Department of Pharmacy, Yokohama City Minato Red Cross Hospital, Yokohama, Japan; 2 Center for Experiential Pharmacy Practice, School of Pharmacy, Tokyo University of Pharmacy and Life Sciences, Hachioji, Japan

**Keywords:** Postoperative nausea and vomiting (PONV), Pharmacist intervention, Multidisciplinary collaboration, Prophylaxis protocol, Risk assessment, Antiemetics, Perioperative care

## Abstract

**Background:**

Postoperative nausea and vomiting (PONV) is a common surgical complication that delays recovery and increases healthcare costs. Although U.S. guidelines recommend multimodal, risk-stratified prophylaxis, Japan lacks unified national guidelines, resulting in practice variation. Despite expanded insurance coverage for 5-HT₃ receptor antagonists, sustained adherence to best practices requires a multidisciplinary framework. We implemented a pharmacist-led, multidisciplinary PONV prevention protocol and evaluated its impact on PONV incidence among adults undergoing elective surgery across multiple specialties.

**Methods:**

We developed a pharmacist-led multidisciplinary PONV prevention protocol based on U.S. guideline algorithms, recommending ≥ 2 and ≥3 prophylactic agents for medium- and high-risk patients, respectively. Pharmacists in the admission support center collected preoperative risk factors, while operating room pharmacists relayed assessments to anesthesiologists and nurses. We conducted a retrospective observational study of patients aged ≥ 18 years undergoing elective gastrointestinal or gynecological surgery under general anesthesia. We compared 110 and 255 patients in the pre- and post-intervention groups, respectively, focusing on patients at medium to high risk. For primary analysis, we performed 1:1 propensity score matching (PSM) using five covariates to control for confounding. Conditional logistic regression evaluated intervention effects while accounting for the matched design.

**Results:**

The final analysis included 106 controls and 243 intervention patients. PSM produced 103 well-balanced matched pairs (standardized differences < 0.1). Conditional logistic regression showed that the pharmacist-led intervention significantly reduced PONV occurrence (odds ratio [OR] 0.308, 95% confidence interval [CI], 0.139–0.680). Prophylactic agent use increased significantly after the intervention (OR 3.95, 95% CI 2.43–6.54), with dexamethasone (OR 5.92, 95% CI 3.03–11.60) and 5-HT₃ receptor antagonists (OR 4.98, 95% CI 2.69–9.22) showing the largest increases. Dopamine antagonists and total intravenous anesthesia administration also showed upward trends, though without statistical significance.

**Conclusions:**

The implementation of a pharmacist-led multidisciplinary PONV prevention protocol was associated with a lower incidence of PONV in medium- to high-risk patients. Pharmacist-conducted preoperative risk assessment improved the implementation of risk-based prophylaxis within a multidisciplinary framework. These findings suggest that pharmacist-driven collaborative approaches may facilitate more consistent PONV prophylaxis in clinical settings lacking unified guidelines.

**Supplementary Information:**

The online version contains supplementary material available at 10.1186/s40780-026-00590-2.

## Background

Postoperative nausea and vomiting (PONV) is one of the most prevalent complications among patients undergoing surgery under general anesthesia. It not only impairs patient comfort [1] but also contributes to delayed postoperative recovery and increased healthcare costs [2]. The incidence of PONV remains highly variable depending on the type of surgery, anesthetic technique, and individual patient risk factors, with reported rates as high as 80% in high-risk patients [3]. According to the *Fourth Consensus Guidelines for the Management of Postoperative Nausea and Vomiting* issued by the Society for Ambulatory Anesthesia (U.S. PONV guidelines), multiple prophylactic interventions are recommended when at least one risk factor is present, emphasizing the necessity of risk-stratified prophylaxis [4]. The U.S. PONV guidelines present 5-HT_3_ receptor antagonists as a standard prophylactic option; the insurance coverage of these agents was expanded in Japan in 2021, potentially promoting their use in PONV prevention.

In Japan, national guidelines for PONV have not yet been established, and clinical practices often vary between institutions and within individual facilities. Even when international guidelines are accessible, their consistent application in high-volume perioperative settings can be challenging. Systematic risk stratification and documentation for PONV are frequently difficult to integrate uniformly into routine anesthetic workflows without a dedicated multidisciplinary framework.

To address these challenges, pharmacist involvement in perioperative care has emerged as a potential strategy to support the implementation of risk-based PONV prophylaxis. At our institution, operating room pharmacists have performed various roles since April 2016, including medication management, protocol development, preoperative pharmaceutical care in collaboration with the Admission and Discharge Support Center and ward pharmacists, and participation in postoperative pain management teams. Previous studies have reported that pharmacist-driven recommendations for PONV prophylaxis can influence PONV incidence [5] and that pharmacist-led risk assessment and prophylactic suggestions based on intraoperative management criteria can reduce PONV occurrence [6], suggesting that pharmacists may contribute to improved PONV prevention. However, prior research has generally focused on specific populations, such as women or patients undergoing gynecologic surgery [7, 8], with studies including multiple surgical specialties and both sexes remaining limited. Moreover, many previous investigations relied on simple between-group comparisons without adequately adjusting for differences in patient characteristics. Evidence evaluating pharmacist-involved prophylactic interventions for PONV following the expanded insurance coverage of 5-HT_3_ receptor antagonists is also scarce. Although sustained system-based implementation has been identified as essential for achieving guideline adherence [9], sustainable PONV prevention systems grounded in multidisciplinary collaboration have yet to be thoroughly examined. These challenges highlight the potential value of pharmacist-led standardized risk assessment and structured information sharing as a collaborative task shifting and sharing approach, in which systematic risk assessment processes can be shared to complement anesthetic decision-making.

In this study, we evaluated the impact of pharmacist-led intervention on PONV prophylaxis within a routine multidisciplinary practice among patients undergoing gastrointestinal or gynecological surgery under general anesthesia, encompassing both sexes. Admission and Discharge Support Center pharmacists and operating room pharmacists established a standardized preoperative risk assessment process and enhanced information sharing with anesthesiologists and operating room nurses.

## Methods

### Study design and settings

This was a retrospective observational study conducted at Yokohama Minato Red Cross Hospital to evaluate the impact of a pharmacist-led, multidisciplinary PONV prophylaxis protocol on clinical outcomes.

### Protocol development

A multidisciplinary PONV prophylaxis protocol was developed by operating room pharmacists and approved by the hospital’s Operating Room Management Committee. The protocol was based on an algorithm presented in the U.S. PONV guidelines [4] and stratified patients according to the number of risk factors present. The risk factors defined in the protocol included female sex, age < 50 years, non-smoking status, history of PONV and/or motion sickness, type of surgery (laparoscopic, gynecological, or cholecystectomy), and postoperative opioid use (Supplementary materials; Table S1).

The protocol recommended the administration of two prophylactic agents for patients with one or two risk factors and three or more agents for those with three or more risk factors. To implement the protocol, pharmacists at the Admission and Discharge Support Center assessed the risk factors before admission, while operating room pharmacists subsequently scored the risk preoperatively, establishing a workflow to recommend appropriate prophylaxis based on the stratified risk.

The recommended prophylactic agents included 5-HT₃ receptor antagonists, dopamine antagonists (droperidol or metoclopramide), dexamethasone, and propofol-based total intravenous anesthesia (TIVA), all of which were available in the operating room. Details regarding the dosage and timing of administration for each agent are provided in Supplementary materials (Table S2). During the study period, the specific 5-HT₃ receptor antagonist used at our institution was changed because of formulary revisions: ondansetron was used from July 2022 to September 10, 2022, followed by granisetron from September 11, 2022, to February 2023.

**Fig. 1:**
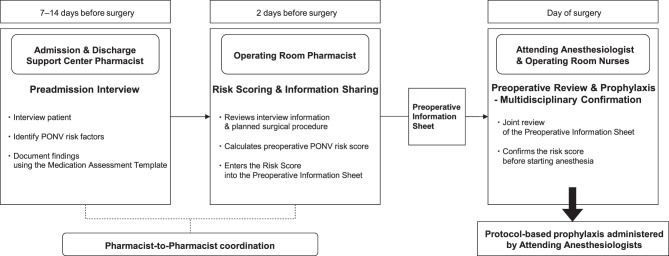
Multidisciplinary perioperative workflow for PONV risk assessment and protocol-based prophylaxis

The pharmacists at the Admission and Discharge Support Center conducted interviews 7–14 days before surgery using the Medication Assessment Template embedded in the electronic medical record system (HOPE LifeMark-HX, Fujitsu Japan Ltd.) to document risk factors. Thereafter, the operating room pharmacists calculated the PONV risk score 2 days before surgery and recorded the results on the patient’s preoperative information sheet. On the day of surgery, the attending anesthesiologists and operating room nurses reviewed this information before anesthesia induction and selected prophylactic agents according to the patient’s risk category (Fig. 1). The final decision regarding the selection of prophylactic medications and use of postoperative opioids was made by the anesthesiologist.

### Patients and data collection

The study population comprised 365 patients (aged ≥ 18 years) who underwent elective gastrointestinal or gynecological surgery under general anesthesia at our institution between July 2022 and February 2023. Patients who underwent surgery between July and September 2022 constituted the pre-intervention control group (*n* = 110), whereas those who underwent surgery between September 2022 and February 2023 constituted the post-intervention group (*n* = 255).

Patients undergoing emergency procedures or day surgeries were excluded because they did not undergo preoperative assessment by the Admission and Discharge Support Center pharmacists and were therefore ineligible for the protocol. Patients undergoing hepatic resection were also excluded because methylprednisolone, which is known to exert PONV preventive effects [4], is routinely administered in such cases.

### Outcomes and variables

The primary outcome was the incidence of PONV, defined as the occurrence of nausea or vomiting within 24 h after surgery. The outcome was considered positive if any of the following were documented in the medical records (anesthesia records, postoperative rounds, pharmacist notes, or nursing documentation): administration of antiemetic medication, recorded observations of nausea or vomiting, or patient-reported symptoms such as “nausea,” “feeling sick,” “vomiting,” “queasiness,” or equivalent expressions.

Data regarding patient characteristics and intraoperative management were retrieved from medical records. Preoperative risk factors present at the time of surgery, including female sex, age < 50 years, non-smoking status, history of PONV and/or motion sickness, type of surgery (laparoscopic, gynecological, or cholecystectomy), and postoperative opioid use, were assessed for each patient.

Other collected variables included intraoperative prophylactic medications and exposure to TIVA. Based on the risk factors defined in the protocol, patients were classified into medium- (1–2 factors) and high-risk (≥3 factors) groups. Low-risk patients were excluded from the analytical cohort because they were not candidates for prophylactic recommendations.

### Statistical analysis

For primary analysis, propensity score matching (PSM) was performed to compare the incidence of PONV between the pharmacist intervention and non-intervention groups. Propensity scores were calculated using a logistic regression model that included the following covariates: American Society of Anesthesiologists physical status (ASA-PS), body mass index (BMI), PONV risk group (medium vs. high), duration of anesthesia, and use of epidural anesthesia. Restricted cubic spline terms with four knots were incorporated for the duration of anesthesia to model its potential non-linear relationship with treatment assignment [10]. Knots were placed at standard percentiles of the distribution. One-to-one nearest-neighbor matching without replacement was conducted using a caliper width of 0.2 standard deviations of the propensity score. Covariate balance was assessed using standardized mean differences (SMDs), with an SMD < 0.1 indicating acceptable balance.

Before matching, the baseline characteristics, PONV risk categories, PONV incidence, and prophylactic medication use were compared between the groups using Fisher’s exact test. After matching, the association between pharmacist intervention and PONV incidence was evaluated using conditional logistic regression, and odds ratios (ORs) with 95% confidence intervals (CIs) were calculated.

During secondary analyses, factors associated with PONV incidence were examined using multivariate logistic regression, and factors influencing the number of prophylactic agents administered were explored using ordinal logistic regression.

All statistical analyses were performed using EZR version 1.61, with two-sided *P* values < 0.05 considered statistically significant.

## Results

**Fig. 2:**
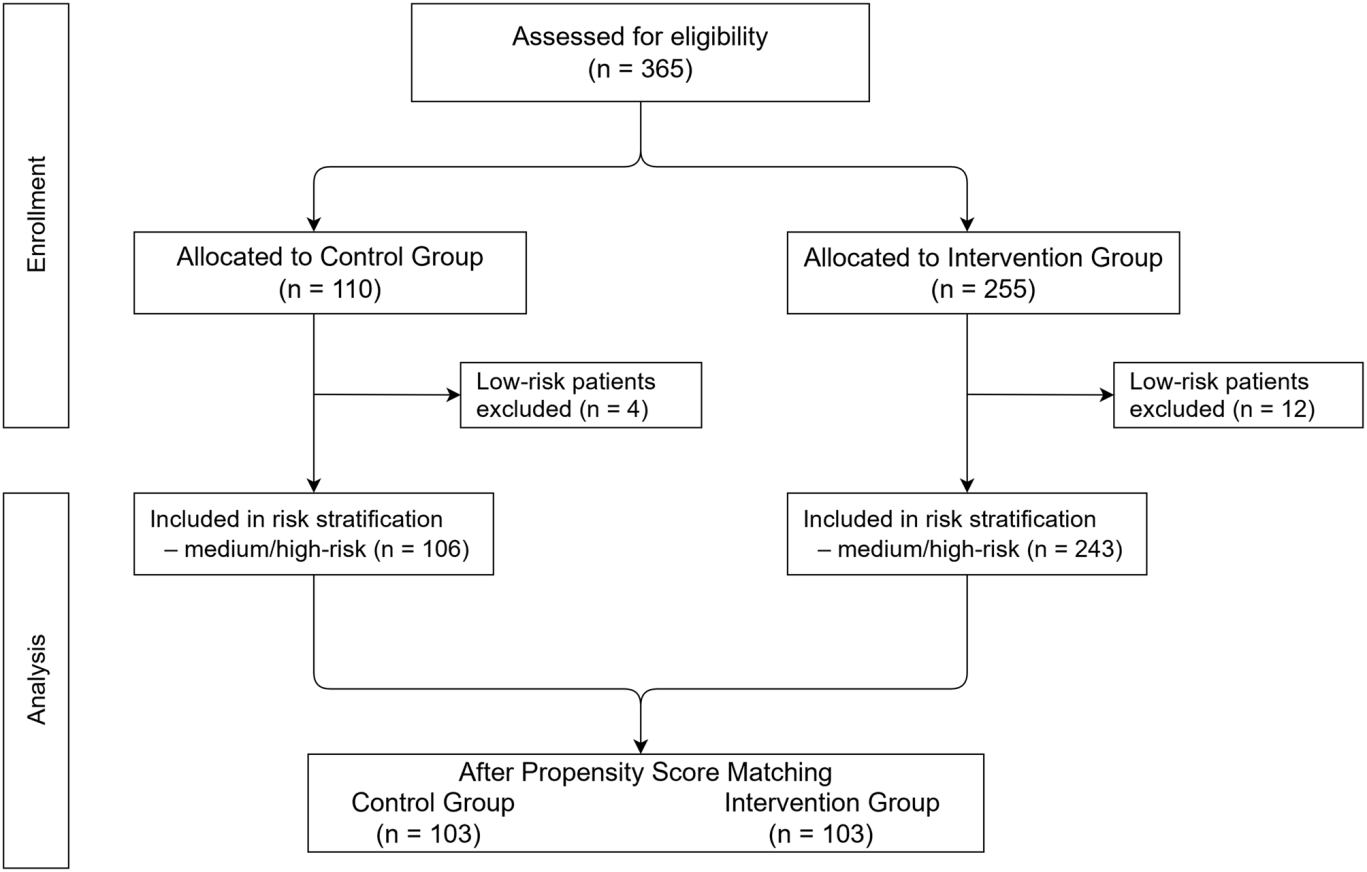
Patient flow diagram showing the selection of medium/high-risk patients and formation of the control and intervention groups before and after propensity score matching

A total of 349 patients were included in the analytical cohort: 106 in the control group and 243 in the intervention group (Fig. 2). Before PSM, the distribution of PONV risk categories was comparable between the groups; the proportions of medium- and high-risk patients were 40.0% and 56.4% in the control group, and 39.2% and 56.1% in the intervention group, respectively, with no significant differences (Supplementary materials; Table S3).

Regarding postoperative management, a standardized protocol for opioid-based analgesia, such as patient-controlled analgesia (PCA), had not been established at our institution during the study period. Postoperative opioid use was recorded in only four patients in the entire cohort (*n* = 2 in the control group and *n* = 2 in the intervention group). Droperidol administration, whether as a single intravenous bolus or via a postoperative PCA regimen, was performed by an anesthesiologist in the operating room. Therefore, these doses were fully captured and recorded as intraoperative administration in our dataset (Supplementary materials; Table 1). No additional droperidol was administered after the patient left the operating room during the 24-hour observation period.

To adjust for confounding factors between the groups, PSM was performed, resulting in 103 matched pairs (Table 1). After matching, adequate covariate balance was achieved, with all SMDs < 0.1.

**Table 1:** Comparison of baseline characteristics before and after propensity score matching

Variable		Before Propensity Score Matching		SMD	After Propensity Score Matching		SMD
		Control Group (*n* = 106)	Intervention Group (*n* = 243)		Control Group (*n* = 103)	Intervention Group (*n* = 103)	
PONV Risk Categories, n (%)							
	High	62 (58.5)	143 (58.8)	0.007	61 (59.2)	60 (58.3)	0.020
	Medium	44 (41.5)	100 (41.2)		42 (40.8)	43 (41.7)	
ASA-PS, n (%)							
	1	23 (21.7)	73 (30.0)	0.212	23 (22.3)	24 (23.3)	0.096
	2	70 (66.0)	139 (57.2)		68 (66.0)	64 (62.1)	
	3	12 (11.3)	30 (12.3)		12 (11.7)	15 (14.6)	
	4	1 (0.9)	1 (0.4)				
BMI, mean (SD)		23.49 (4.05)	23.33 (3.73)	0.041	23.42 (4.04)	23.38 (3.55)	0.011
Duration of Anesthesia (min), median (IQR)		184 (78–707)	179 (57–841)	0.061	184 (80–707)	173 (57–690)	0.050
Epidural Anesthesia, n (%)	No	33 (31.1)	77 (31.7)	0.012	32 (31.1)	33 (32.0)	0.021
□	Yes	73 (68.9)	166 (68.3)	□	71 (68.9)	70 (68.0)	□

SMD: Standardized Mean Difference, SD: Standard Deviation, IQR: Interquartile Range

For the conditional logistic regression analysis following PSM, the pharmacist’s intervention was associated with lower odds of developing PONV, with an OR of 0.308 (95% CI, 0.139–0.680) (Table 2).

**Table 2:** Primary analysis of PONV incidence between the pharmacist intervention and control groups after propensity score matching

Outcome	Control group (n = 103)	Intervention group (n = 103)	Odds ratio	95% CI
PONV occurrence, *n* = 206	33 (32.0)	15 (14.6)	0.308	(0.139–0.680)

CI: Confidence Interval

The OR for receiving multimodal prophylaxis in the intervention group was 3.95 (95% CI, 2.43–6.54) compared with the control group (Table 3). Table 3 summarizes the use of prophylactic medications. Following intervention by pharmacists, the ORs for the use of dexamethasone and 5-HT_3_ receptor antagonists were 5.92 (95% CI, 3.03–11.60) and 4.98 (95% CI, 2.69–9.22), respectively. Although the use of dopamine antagonists and propofol-based TIVA increased after the intervention, these changes were not statistically significant.

**Table 3:** Subgroup analysis of multivariate association between PONV prophylaxis and associated factors before propensity score matching

Outcomes	Variable	Odds Ratio	95% CI	P
Multimodal Prophylaxis	Pharmacist Intervention	3.95	(2.43–6.54)	<0.001
□	Duration of Anesthesia	1.00	(0.998–1.000)	0.833
□	ASA-PS	0.48	(0.331–0.696)	<0.001
□	BMI	0.97	(0.916–1.020)	0.246
□	Epidural Anesthesia	1.34	(0.815–2.200)	0.252
□	Medium Risk (vs. High Risk)	0.12	(0.0724–0.207)	<0.001
Dexamethasone	Pharmacist Intervention	5.92	(3.03–11.60)	<0.001
□	Duration of Anesthesia	1.00	(0.996–1.000)	0.336
□	ASA-PS	0.73	(0.470–1.120)	0.148
□	BMI	0.97	(0.907–1.040)	0.398
□	Epidural Anesthesia	1.42	(0.776–2.600)	0.256
□	Medium Risk (vs. High Risk)	0.22	(0.119–0.403)	<0.001
5-HT_3_ receptor antagonists	Pharmacist Intervention	4.98	(2.69–9.22)	<0.001
□	Duration of Anesthesia	1.00	(0.999–1.000)	0.197
□	ASA-PS	0.56	(0.358–0.866)	0.009
□	BMI	1.00	(0.933–1.070)	0.951
□	Epidural Anesthesia	0.99	(0.543–1.810)	0.979
□	Medium Risk (vs. High Risk)	0.14	(0.0745–0.258)	<0.001
TIVA	Pharmacist Intervention	1.19	(0.659–2.170)	0.558
□	Duration of Anesthesia	1.00	(0.991–0.999)	0.009
□	ASA-PS	0.43	(0.266–0.699)	<0.001
□	BMI	0.97	(0.898–1.040)	0.354
□	Epidural Anesthesia	1.62	(0.837–3.120)	0.153
□	Medium Risk (vs. High Risk)	0.14	(0.0646–0.300)	<0.001
Dopamine antagonists	Pharmacist Intervention	1.20	(0.505–2.830)	0.684
□	Duration of Anesthesia	1.00	(0.999–1.010)	0.157
□	ASA-PS	0.51	(0.255–1.040)	0.063
□	BMI	0.93	(0.839–1.040)	0.201
	Epidural Anesthesia	1.88	(0.660–5.380)	0.237
□	Medium Risk (vs. High Risk)	0.39	(0.140–1.090)	0.072

## Discussion

This study demonstrates that the implementation of an intraoperative PONV prophylaxis protocol through pharmacist involvement promoted risk category-based prophylaxis and improved adherence to guideline-concordant antiemetic administration. Following the intervention, an increased proportion of patients in the medium- and high-risk categories received appropriate prophylaxis. After PSM, a lower incidence of PONV was observed in the intervention group. These findings suggest an association between pharmacist intervention and reduced PONV incidence, likely mediated through enhanced implementation of risk-based prophylactic antiemetic use.

We conducted PSM analyses to address the potential confounding factors inherent in retrospective studies. This approach enabled the statistical balancing of patient demographics and surgery-related variables, allowing for a more rigorous evaluation of the association between pharmacist intervention and the incidence of PONV. In particular, anesthesia duration was modeled using restricted cubic splines to appropriately capture its non-linear relationship with treatment assignment. Given that anesthesia duration is strongly associated with both treatment allocation and PONV risk, appropriate modeling of this variable was considered essential. As a result, adequate covariate balance was achieved across all evaluated variables, with all SMDs falling below the conventional threshold of 0.1, supporting the validity of the matched comparison. Furthermore, the consistent beneficial effect of the pharmacist-led intervention across subgroups with different risk profiles, such as sex and surgical department (gynecology or gastrointestinal surgery), supports the robustness of the observed association. These findings suggest that the intervention may be applicable across diverse patient groups within the studied clinical settings. Given that the study period overlapped with the widespread availability of 5-HT₃ receptor antagonists in Japan, the findings also provide practical evidence that reflects current clinical practice.

The present analyses clearly demonstrated the contribution of pharmacist interventions to both prophylaxis rates and PONV incidence. As the intervention specifically targeted medium- to high-risk patients, analyses excluding low-risk patients represented the most appropriate assessment of its effectiveness. Even in this analysis, the incidence of PONV was significantly lower in the intervention group, confirming the impact of pharmacist involvement in patients at elevated risk. In the high-risk category, the increased use of three or more prophylactic agents corresponded to a significant reduction in the incidence of PONV, suggesting that pharmacist intervention contributed to risk-stratified prophylaxis. These observations were consistent with the results of the stratified analyses using Fisher’s exact test and descriptive statistics. Although the proportion of medium-risk patients receiving two or more agents did not differ significantly, the rate of no prophylaxis significantly decreased, and the administration of at least one prophylactic agent significantly increased following the intervention. These findings indicate a shift from underutilization toward more proactive prophylactic management in medium-risk patients, although full adherence to the multi-agent regimen was not achieved. Thus, while complete adherence remained limited, the overall direction of change supports the interpretation that pharmacist intervention promoted incremental and risk-aware optimization of prophylaxis.

Among prophylactic medications, the use of dexamethasone and 5-HT₃ receptor antagonists significantly increased after pharmacist intervention. As both are strongly recommended in the U.S. PONV guidelines, protocol implementation likely promoted the standardized selection of these agents. Regarding 5-HT₃ receptor antagonists, this trend may be partly attributed to the expansion of insurance coverage in Japan, which has significantly reduced barriers to their clinical use. However, it should be noted that dexamethasone and droperidol currently lack official insurance indications for PONV in Japan. While our pharmacist-led protocol established an institutional consensus that facilitated the standardized use of these medications, their off-label status may still have influenced the prioritization of drug selection by anesthesiologists. Multivariate analyses further showed that higher ASA-PS scores were negatively associated with the selection of 5-HT₃ receptor antagonists and TIVA, suggesting that younger patients with a better physical status and higher PONV risk were more likely to receive these preventive strategies. In contrast, the use of TIVA decreased as the duration of anesthesia increased, potentially reflecting practical constraints, such as equipment management and the complexity of drug handling. Dopamine antagonists may have been used selectively because of concerns regarding extrapyramidal symptoms; however, collaboration with the Acute Pain Service, which includes operating room pharmacists, may facilitate safer use and enhanced monitoring of adverse effects where appropriate.

This study has several limitations. First, as a single-center retrospective study employing a historical control design, mild PONV episodes may have been underreported in the medical records. In addition, the absence of a contemporaneous control group makes it difficult to fully disentangle the effect of the pharmacist intervention from natural temporal changes, such as anesthesiologists becoming more familiar with the institutional protocol over time (i.e., a learning effect). Second, the limited sample size may have reduced the ability to detect statistically significant differences in some analyses. Third, although adequate covariate balance was achieved for the measured variables using PSM, residual confounding by unmeasured factors cannot be completely excluded. Postoperative opioid use is a well-known risk factor for PONV; however, in this study, the frequency of postoperative opioid administration was extremely low. Therefore, its influence on the overall incidence of PONV and the study’s conclusions is likely minimal. Moreover, we did not explicitly model the increased use of prophylactic antiemetics as either a mediator or a confounder in the causal pathway. Therefore, the causal interpretation of the association between pharmacist involvement and reduced PONV incidence should be made with caution.

Despite these limitations, this study is one of the few reports to statistically demonstrate the utility of guideline-based PONV prophylaxis protocols and pharmacist intervention using real-world clinical data. The checklist for perioperative pharmacy services issued by the Japanese Society of Hospital Pharmacists [11] also recommends establishing a prophylactic framework for patients at risk of PONV through collaboration with anesthesiologists and surgical units. In our institution, operating room pharmacists played a central role in developing the PONV prophylaxis protocol, standardizing the criteria for prophylactic administration, managing formulary selection, and ensuring medication availability. This organizational approach facilitated appropriate prophylaxis based on the number of risk factors and contributed to improved prophylaxis rates. Such a pharmacist-led system development and multidisciplinary collaboration have enabled sustainable implementation, which is a distinctive feature of the present study. Furthermore, the establishment of the protocol strengthened coordination among preoperative pharmacists, anesthesiologists, and operating room nurses, thereby enhancing the continuum of care from preoperative risk assessment to postoperative follow-up.

## Conclusions

The pharmacist-led intervention incorporating standardized preoperative risk assessment and structured information sharing improved the implementation of risk-based PONV prophylaxis in routine perioperative practice. By systematically integrating risk stratification into the workflow, pharmacists contributed to more consistent prophylactic decision-making across surgical populations.

This approach enabled the establishment of a sustainable model of pharmacist involvement that is not dependent on individual expertise or specific staffing configurations. Through collaborative task shifting and sharing, pharmacists assumed responsibility for risk assessment and information management components, supporting anesthesiologists while preserving anesthetic decision-making authority. Such a pharmacist-led framework may contribute to reducing practice variability and advancing sustainable perioperative pharmaceutical care.

## Electronic supplementary material

Below is the link to the electronic supplementary material.

Supplementary Material 1

## Data Availability

The datasets used and/or analyzed in the current study are available from the corresponding author upon reasonable request and subject to institutional data sharing policies.

## Abbreviations

PONV: Postoperative Nausea and Vomiting
PSM: Propensity Score Matching
OR: Odds Ratio
CI: Confidence Interval
TIVA: Total Intravenous Anesthesia
ASA-PS: American Society of Anesthesiologists physical status
BMI: Body Mass Index
SMD: Standardized Mean Difference
PCA: Patient-Controlled Analgesia
SD: Standard Deviation
IQR: Interquartile Range
